# How do medical professionals make sense (or not) of AI? A social-media-based computational grounded theory study and an online survey

**DOI:** 10.1016/j.csbj.2024.02.009

**Published:** 2024-02-17

**Authors:** Sebastian Weber, Marc Wyszynski, Marie Godefroid, Ralf Plattfaut, Bjoern Niehaves

**Affiliations:** a University of Bremen, Digital Public, Bibliothekstr. 1, 28359 Bremen, Germany; b University of Siegen, Information Systems, Kohlbettstr. 15, 57072 Siegen, Germany; c University of Duisburg-Essen, Information Systems and Transformation Management, Universitätsstr. 9, 45141 Essen, Germany

**Keywords:** Artificial intelligence (AI), Medical professionals, Medical AI, Technology acceptance, Job-replacement anxiety, Social media analysis

## Abstract

To investigate opinions and attitudes of medical professionals towards adopting AI-enabled healthcare technologies in their daily business, we used a mixed-methods approach. Study 1 employed a qualitative computational grounded theory approach analyzing 181 Reddit threads in the several subreddits of r/medicine. By utilizing an unsupervised machine learning clustering method, we identified three key themes: (1) consequences of AI, (2) physician–AI relationship, and (3) a proposed way forward. In particular Reddit posts related to the first two themes indicated that the medical professionals' fear of being replaced by AI and skepticism toward AI played a major role in the argumentations. Moreover, the results suggest that this fear is driven by little or moderate knowledge about AI. Posts related to the third theme focused on factual discussions about how AI and medicine have to be designed to become broadly adopted in health care. Study 2 quantitatively examined the relationship between the fear of AI, knowledge about AI, and medical professionals' intention to use AI-enabled technologies in more detail. Results based on a sample of 223 medical professionals who participated in the online survey revealed that the intention to use AI technologies increases with increasing knowledge about AI and that this effect is moderated by the fear of being replaced by AI.

## Graphical Abstract

**Figure:**
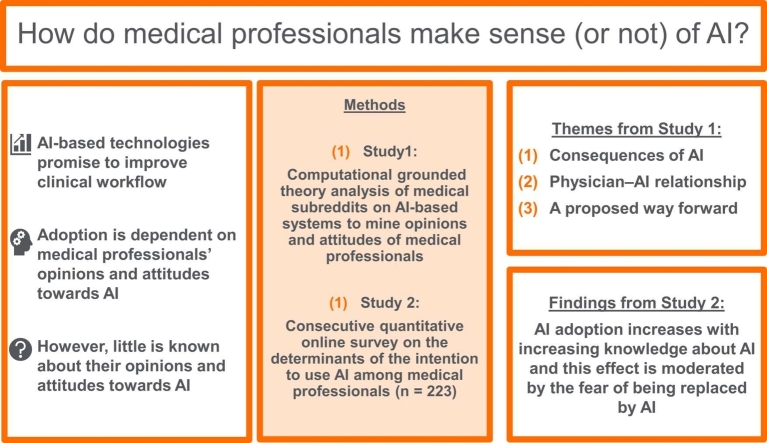

## 1 Introduction

Healthcare systems across the globe are currently struggling with increasing demands to provide patient-centered, innovative, comprehensive care. Moreover, demographic changes as well as the accompanying shortages of medical professionals are simultaneously inducing an undersupply of healthcare in addition to creating challenging working conditions [25], [79]. Digital technologies promise to address these current issues in healthcare by improving clinical workflows and care processes in order to increase patient safety, treatment efficiency, and quality of care [58]. For example, the increasing volume of health-related data collected by healthcare organizations – such as primary care practices, hospitals, and health insurance companies – has already become more and more valuable and usable in the healthcare domain [83] and it is a useful resource that, e.g., can be applied to improve medical decision-making processes. However, individuals usually have limited cognitive resources, leaving them unable to process such large amounts of information when making decisions. Cognitive limitations may constrain peoples’ rationality, which can result in sub-optimal or biased choices (bounded rationality; [77], [78]) potentially having serious consequences. One way to incorporate a large amount of data into medical decision-making processes is using clinical decision support systems based on AI-algorithms. In this regard, research has shown that AI – with its ability to process and learn from large datasets – can play an important role in various clinical tasks [90], such as diagnosis [91], treatment [24], and clinical decision-making [10].

Implementing AI technology in clinical tasks to improve performance can only be successful if medical professionals, i.e., those who are supposed to use the technology, are involved in the implementation process. But only a few studies have investigated opinions and attitudes of medical professionals toward AI technology and their intention to adopt it or related research topics so far. In this context, most studies focus on attitudes towards usefulness, job-replacement anxiety, and trust in automation. In particular, Oh et al. [61] found that medical professionals consider AI technology to be useful, especially in diagnosis and treatment planning. Furthermore, two thirds of their respondents did not rule out the possibility of their job being replaced by AI. Another study found that at least one third of healthcare practitioners consider AI to be likely to replace physicians in specific key tasks (i.e., diagnoses, prognoses, referral, treatment plans, documentation; [15]). In particular in areas in which AI already performs better than humans, some medical professionals fear that they could be replaced by the new technology [1], [33], [61], with the intensity of this job-replacement anxiety increasing with the physicians’ level of knowledge about AI [1], [40]. Furthermore, other research brings into play the relationship between trust in AI-based healthcare systems and the intention to use them, with trust in AI being influenced by several factors such as complexity of algorithms, cognitive biases, and AI knowledge [8].

However, little is known on whether or not medical professionals make sense of using AI within their domain. The subjective understanding of AI by medical professionals, their general opinions and attitudes towards AI and its adoption into clinical practice, i.e., their intention to use AI-based systems, are still unclear. In this regard, other authors also plea for a further in-depth exploration [14] as it may provide important information of how AI can improve performance in healthcare. Therefore, we propose the following research question (RQ):

*RQ: What do medical professionals think about adopting AI-enabled healthcare technologies?*

To answer the RQ, a mixed-method approach was employed. As more and more medical professionals are using online information [56], we crawled posts and comments from medical subreddits (strictly moderated for professional content) on the social media platform Reddit in order to analyze characteristic content and patterns concerning (potential) human–AI collaboration. In addition, we conducted a quantitative study using an online survey, to investigate the impact of AI-induced anxiety and knowledge about AI on intention to use AI-based systems among medical professionals.

## 2 Theoretical background

Digital technologies are used to support and accelerate the work routines of medical professionals by helping them handle growing workloads and case complexities. Along with the introduction of AI technologies, the technologies themselves are also currently evolving. For the present text, the term “AI” is used to refer to technologies that require capabilities that lie at the “frontier of computational advancements [and] that reference […] human intelligence in addressing ever more complex decision-making problems” ([11], p. 1435). AI promises to increase performance and decrease the effort of active use in various areas. For example, clinical decision support systems (CDSSs) are utilized to predict adverse drug effects [63] or unplanned hospital re-admission [62] or to assist medical professionals in finding suitable diagnoses [6]. By integrating AI techniques into these CDSSs, both performance and reliability increase [80]. Nevertheless, the purposes and functions of AI strongly differ between contexts of use. While AI-enabled conversational agents using natural language processing (NLP) are aiming to support patients with chronic diseases [21], [74], [88], AI is also utilized to improve skin cancer diagnosis through machine learning algorithms that use image recognition [31]. Furthermore also the maturity of AI solutions varies [6], [74] depending on the current state in the AI lifecycle. The AI lifecycle describes a possibly recurring cycle of development which ranges from initial planning (i.e., establishing clear expectations of the solution and perform data selection, model training, and validation) to a deployed solution that is integrated into medical practice using live data and getting monitored to evaluate its performance to take further possible development decisions to ensure safety and effectiveness [30].

Many people, lay persons as well as experts from various fields, including medical professionals, lack in a comprehensive understanding of AI, technical knowledge, and its versatile potential, which might be due to a limited experience in using AI [20], [5]. Hence, they still know little about what and how AI can change their own routines and practice, although many of these professionals appear to anticipate that AI-enabled technologies can save time or improve monitoring [50]. Indeed, Laï et al. [50] revealed that the most medical professionals view AI as an autonomous entity rather than as a concept that can be integrated into different contexts and technologies, which is, however, not surprising, reflecting on the plethora of application domains and terms used to describe AI-based systems [27]. Therefore, it is conceivable that medical professionals generalize their specific perceptions and transfer them to other contexts in which they encounter or interact with AI-enabled technologies (or will do so in the future). For instance, there appear to be contrary views on AI-enabled advice. Some experts are skeptical about such advice, whereas others are overly confident and unconditionally trust it. Studies have shown both that highly experienced medical professionals rate advice from AI as being worse than advice from humans, independent of the quality of the advice, and that less-experienced medical professionals sometimes favor an incorrect AI decision, even if their own initial diagnosis was correct [34], [44].

Furthermore, drivers such as fear create certain preconceptions about AI, particularly in domains in which AI already outperforms humans at certain tasks. In radiology, for example, 38% of medical personnel are concerned about being replaced by AI [40]. Interestingly, however, this fear seems to be related to the level of knowledge about AI, with the fear of being replaced by an AI decreasing with increasing specific knowledge about AI systems. Moreover, individuals with greater AI knowledge seem to have more positive attitudes towards AI-enabled technologies [40]. Against this background, research has recently begun to shift its focus to strategies for studying medical professionals’ perceptions of AI [1], [61], [76], [92].

Along with this shift, questions concerning the determinants of accepting AI-enabled systems, the willingness to adopt AI into practice, and the intention to use AI-based technologies have come to the fore [27]. Research on the impact of human-like augmentation of technologies on the adoption of these technologies indicates that psychological factors are becoming increasingly relevant in human–AI collaboration research, whereas other factors that have traditionally explained and predicted technology adoption – such as ease-of-use (also called “effort expectancy”) – have become less relevant [75], [92]. In particular, recent research suggests that psychological traits, e.g. personality, attitudes, behavior styles, and other individual characteristic may have significant effects on the sustainable and successful collaboration between humans and AI-enabled technology [18], [31]. Therefore, medical professionals’ opinions and attitudes towards AI are of great interest to research on the adoption of AI-enabled systems. As Tschandl et al. Tschandl et al., [80] suggest, among medical professionals, distinct types or groups of users likely exist whose opinions and attitudes towards AI differ, for example, due to each individual’s knowledge in terms of medical and technological expertise. Both uncovering and distinguishing between these different views of AI that are held by certain groups appear to be critical to the long-term implementation and adoption of AI-enabled technologies in healthcare in the near future [20]. Therefore, we aimed to shed light both on the different perceptions and connotations that medical professionals have regarding AI as well as on the possible impact that these perceptions and connotations may have on technology adoption.

## 3 Study 1

To investigate medical professionals’ thoughts about using AI health care technologies, we combined a qualitative research approach with quantitative elements. We retrieved statements from medical professionals about AI from specific channels of the social media platform Reddit and analyzed it using a social-media-based computational grounded theory approach [59].

### 3.1 Method

#### 3.1.1 Selection of subreddits

Founded and published in 2005 [68], Reddit is a social media platform that serves as a social news aggregator and that contains discussions on various topics, including web content ratings. The platform allows registered users to publish content in the form of text posts, links, pictures, and so on, and organizes these posts by subject in so-called “subreddits”. The interaction of Reddit’s users (called “redditors”) is designed such that they can downvote or upvote posts, comment on posts, and respond in a conversation tree of comments (so-called threads). Since its creation, the platform has been growing continuously. By the end of 2020, Reddit had 52 million daily active users who had contributed 303.4 million posts, 2 billion comments, and 49.2 billion upvotes within the year [67].

As our goal was to investigate the perceptions that medical professionals have of AI, we focused on the medical subreddits of r/medicine, r/radiology, r/surgery, and r/psychiatry. The subreddit of r/medicine is one of the largest medical subreddits. To cover a broad and diversified spectrum of opinions and attitudes towards AI ant its adoption into clinical practice, we additionally included related subreddits that focus on physician–patient interactions (i.e., r/psychiatry) and on technology usage (i.e., r/radiology and r/surgery). In general, these subreddits describe themselves as communities of medical professionals who discuss related topics. Table 1 presents the self-descriptions of each community and each community’s current number of members. Specific community rules (e.g., no asking for medical advice, and no marketing) regulate the discussions within the subreddits to maintain the quality of discourse. The channels are supervised by moderators who have the authority to enforce these rules. Reddit can thus be viewed as platform on which medical professionals discuss recent medical topics, and the platform thereby serves as a valid source for collecting relevant information about the perceptions that medical professionals have of AI.

**Table 1:** Description of subreddits.

Subreddit	Self-description	Members	Created
r/medicine	A virtual lounge for physicians and other medical professionals from around the world to talk about the latest advances, controversies, ask questions of each other, have a laugh, or share a difficult moment. This is a highly moderated subreddit. Please read the rules carefully before posting or commenting.	349,000	2008
r/radiology	We aim to become the reddit home of radiologists, radiographers, technologists, sonographers and lay-users interested in medical imaging.	50,400	2008
r/surgery	*Not provided*	27,600	2008
r/psychiatry	We're a community created for psychiatrists and others in the mental health field to come together and discuss our field. We are not a subreddit to ask psychiatrists questions either about individual situations or about psychiatry generally.	66,700	2009

#### 3.1.2 Research design

**Fig. 1:**
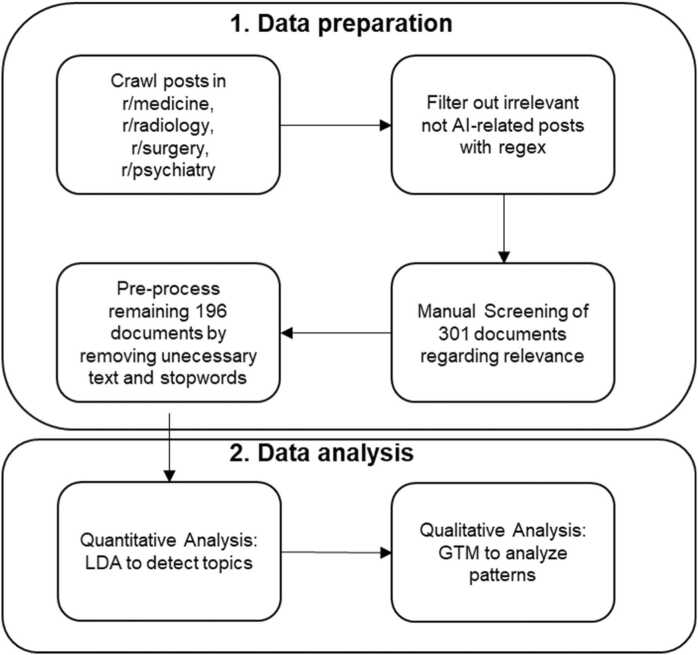
Research process of study 1.

For study 1, we employed an observational study design and used purposive sampling by filtering Reddit threads related to the topic. We applied a two-staged process that included data preparation and data analysis. Data preparation involved the steps of data sampling, collection, and pre-processing. For data analysis, we adapted a computational grounded theory approach that combined “human knowledge and hermeneutic skills with the processing power and pattern recognition of computers” ([59], p. 3). Each Reddit thread served as a unit of analysis. Fig. 1 summarizes the research process, which we describe in detail below.

#### 3.1.3 Data sampling, collection, and pre-processing

We crawled every post in the medical subreddits from 1 January 2015 to 31 December 2022. As the official Reddit API is limited to a few requests, we used the Pushshift API, which collects and archives Reddit data from 2015 to the present and that thereby allows a huge dataset to be gathered [9]. We extracted the following information from each post: id, subreddit, author, author flair (i.e., a description of job function, if provided), title, text, score, number of comments, URL, and time of creation. We exclusively included AI-related content and removed other posts. In so doing, we filtered posts that contained at least one relevant term from the Wikipedia glossary of AI [86] as well as further relevant terms and their abbreviations (e.g., artificial intelligence, explainable artificial intelligence, machine learning, deep learning, unsupervised learning, supervised learning, reinforcement learning, natural language processing, image recognition, or neural network) using regular expressions (i.e., regex). Subsequently, we retrieved all comments including at least one of the terms listed from the posts. We considered each individual post with at least one comment to be a relevant document for analysis. Since the Pushshift archive is not totally comprehensive, we additionally used the Python Reddit API Wrapper [65] to directly extract missing comment information. The combination of these data extraction methods provides an extensive set of historical and recent data. Data were then transferred into a relational matrix with each row representing one post. One column showed the post information and another column the initial post text combined with the entire conversation tree (i.e., the comments) in the correct hierarchical and chronological order. Next, two of the authors manually screened the entire content of each document according to its suitability for analysis. In sum, 301 documents remained for manual screening. A document was excluded if it only contained a reference to an external source (e.g., a survey or research paper; *n* = 58), if it was sarcastic (*n* = 2), if it was an AI developer post with no opinions from medical professionals (*n* = 4), if it was a meta post (*n* = 1), if it was not AI-related (*n* = 21), or if the content had been removed by Reddit or the user (*n* = 19). For final analysis, a sample of 196 documents (avg. length per document: 1036 words) remained. In line with the literature [52], we next pre-processed the data in the following order: tokenization, lowercase text, removal of punctuation and special characters, removal of stop words, and finally, stemming using the Python packages NLTK and spaCy [13], [39] before moving to the data analysis stage.

#### 3.1.4 Data analysis

For data analysis, we applied a computational grounded theory approach [59] including a pattern detection phase, and an interpretive, qualitative refinement and confirmation phase that enabled us to gain a deeper understanding of the data. In the pattern detection phase, we used the unsupervised machine learning approach of Latent Dirichlet Allocation (LDA) to detect topic clusters in the text corpora [16]. The choice of LDA was motivated by its proven efficacy in discovering latent topics in large datasets, as evidenced by its successful application in healthcare research [53], [72]. LDA's ability to model documents as mixtures of topics, where each topic is characterized by a distribution of words, made it particularly suitable for our objective of uncovering underlying themes in the data without prior labeling. This model allowed us to quickly analyze the data based on topic clustering as well as exemplary reading of threads which belonged to a topic. In the interpretive, qualitative refinement and confirmation phase, we used qualitative methods: Within the clusters identified through the LDA approach, we inductively analyzed the data qualitatively using grounded theory methodology (GTM; [23] to gain a deeper understanding of the data. By intuitively assigning in vivo codes to the thread passages, we began with a shared sample (*n* = 21) of independent open coding. Afterward, we set to find a common standard by discussing and resolving pending issues. The remaining sample was then divided into two parts. Each part of the sample was coded separately. The first categories were created by combining relevant open codes whenever feasible. Axial codes were then created by classifying open codes into larger schemes after reading each Reddit thread, thereby resulting in a higher degree of abstraction.

### 3.2 Findings

In the following, we report the results of the pattern detection phase and the interpretive, qualitative refinement and confirmation phase separately.

#### 3.2.1 Pattern detection phase

By testing the most common 11 LDA topic models ranging from 2 to 13 topics, respectively, we found that the coherence model provided by the genism package [69] best fits our data. The coherence measure indicates a high coherence of topics (i.e., a topic’s top *n* words are related to each other). In particular, we used the C_V_ coherence measure for calculating topic coherence, which performs very good when achieving large correlations to human ratings of topic coherence [71]. However, choosing the appropriate number of topics is a balance act when coherence scores are close together. A large number of topics may result in relatively granular and verbose themes, whereas a small number may not properly capture distinct themes. We chose 7 topics to be returned by LDA for this dataset because this number had the highest coherence score and interpretability of topics seemed plausible.

We then discussed the most salient terms for each topic as well as representative Reddit threads for each of the 7 topics (see Appendix A for the most salient terms for each topic). In this regard, we randomly chose 10 threads of each topic (if available, otherwise all available threads were considered) and two of authors then read and discussed the content of the threads as well as the terms to analyze the topically disjunct themes. As the sample contained some topics that were very granular the authors additionally took the 3-topic model in a similar manner into account (see Appendix for the most salient terms for each topic). Based on this procedure, we derived 3 key themes: (1) the consequences of AI, (2) the physician–AI relationship, and (3) a proposed way forward (see Appendix A for the summary).

#### 3.2.2 Qualitative refinement and confirmation phase

For the qualitative analysis, we focus on the three key themes identified in the pattern detection phase, i.e., (1) consequences of AI, (2) physician–AI relationship, and (3) a proposed way forward. For transparency, we assign each redditor a unique ID (e.g. U1) to identify recurring quotes from a redditor in the debates. Table 2 summarizes the resulting coding scheme and the number of related codes.

**Table 2:** Coding scheme and topic descriptions.

Topic	Description	#Codes
Consequences of AI	How AI will change the medical field	1153
Consequences for medical professions	Prospects about how medical professions will evolve with AI	981
Consequences for medical practice	Prospects about how specific medical practices will change with AI	101
Consequences for the patient–physician relationship	Prospects about how patients will accept AI, and related outcomes	71
Physician–AI relationship	Prospects about how physicians and AI will work together (or not) based on the perception of the current state of AI	501
AI abilities	Discussion about AI abilities based on current data availability and task performance in specific fields	102
AI limitations	Discussion about AI limitations based on current data availability, humanness, and tasks	281
AI acceptance	(Non-)acceptance and constituting factors	118
A proposed way forward	What is necessary for human–AI collaboration	264
Requirements for useful AI	Prospects of what the AI of the future will need	91
Physicians’ knowledge about AI	Prospects of physicians’ future requirements	31
Reflections of AI research	Discussions about conducting research in the medical field that employs AI	142

#### 3.2.3 Consequences of AI

Many threads focused on the consequences of AI for medical professionals and their profession. These discussions included suggestions of areas that would either be especially well-suited or not at all suited for automation. The most prominent specialization under discussion was radiology, and exchanges sometimes grew heated:

*[U1]: and yet we still don't have a machine that can accurately read EKGs.Radiologists do a lot more than just read x-rays, so to say [that] they will be replaced is not quite accurate. […]. [U2]: That's because [the] current ECG reading machine is an Algorithm [and is] not AI based. Out of all the fields, Radiology is the easiest to replace with […] AI. [U1]: HA! Do tell how a Radiologist is so easy to replace? [U2]::) don't take it personally. We're just having a discussion [U3]: Can we just stop having posts about this. Or a weekly AI sticky thread. Please.*

Contrary to highly emotional discussions, creative ideas regarding how to utilize AI in medicine emerged and ranged from managing patient-ventilator synchroneity to predicting heart attacks to diabetes treatment. Discussions that focused on specific aspects and details usually remained highly rational and realistic regarding the potential of AI. However, the underlying debate about possibly replacing medical professionals with AI remained ongoing. Nevertheless, discussions tended to become less focused on details and specific applications as emotions rose. In these cases, the fear of being replaced often provoked a reflection about the paradigms and ideals of medicine:

*[U4]: I'm [a] medical student but artificial intelligence scares me […] [regarding the] future of [the] profession. Are we wasting time by studying? [Is] years of studying worth [it]? […] I always fancied medicine. I wanted to become an outstanding physician. This profession was about science, hard[]work, humanity, with social respect at the same time.*

In addition to consequences for the profession of medicine itself, threads also discussed consequences for medical practice, with economic and organizational effects being prominently represented. Most frequently, threads considered AI to be a measure for lowering the costs of care. Simultaneously, users hoped for an increase in the quality of care through AI, for example, by increasing diagnostic accuracy or reducing re-admission rates. Again, comments ranged from very general statements to specific ideas regarding how to improve medical processes. For example:

*[U5]: I think the next big thing we will see is AI prioritization of radiographs [before they are] read by radiologists.*

Moreover, commenters expressed the expectation that AI would radically affect the patient–physician relationship. While medical professionals did discuss their own acceptance of AI, they also intensely argued about patients’ acceptance of AI. In these arguments, the medical professionals assumed that their patients would not accept AI due to trust issues, or the lack of AI accountability, or the ability of AI to explain how it reaches decisions. In general, medical professionals thought that patients needed a person as a primary point of contact in emergency situations, such as surgery. This argument was often interlinked with the replacement discussion and highlighted patients’ possible resistance to the use of AI as a glimmer of hope:

*[U6]: Yes, I know we will probably come to a place where a machine can do everything I do and do it better and consistently good. But I don't think we are [only] a few years away from that happening[,] as some think, and I think […] patients[, in particular,] will fight it.*

A frequently discussed aspect of possible consequences also involved the interaction between patients and AI. Medical professionals were worried that AI that relies solely on patients’ statements could lead to cause adverse outcomes for these patients. Nevertheless, the discussions highlighted the notion that a substantial prerequisite for desirable patient outcomes rests upon “human” elements, such as empathy and the personal dialogue between physician and patient.

#### 3.2.4 Physician–AI relationship

This category involves statements about AI abilities, limitations, and AI research, in the context of (non-)acceptance of AI. Most prominent across the discussion threads were opinions about AI limitations, which included technical issues, the inferiority of AI to medical professionals, and requirements an AI needs to meet before adopting it into clinical tasks. The technical issues that were mentioned were mostly related to the scalability of current “academic” applications of medical AI and to the lack of technological infrastructure in the current landscape:

*[U7]: As others have mentioned, however, more data, extensive validation, and vast improvements in health informatics infrastructure are required before the routine, widespread implementation of these kinds of models.*

Finally, many medical professionals highlighted the notion that AI is limited by the currently available data. These medical professionals assumed that the quality of the clinical data is insufficient, that patients provide biased data, or that AI is simply unable to communicate with medical professionals in an adequate manner. Some posts discussed the resistance of medical professionals to working together with AI due to such beliefs:

*[U8]: I do image analysis on medical imagining. The lag behind AI for pathology is because pathologists have done a good job of poor data management and [of] not making imagining data accessible.*

However, in the context of attitudes towards AI abilities, we found the exact opposite: Individuals argued that natural language processing would allow AI to provide clinical documentation, that a bit of garbled data would not hinder progress, and that studies had shown that sufficient data for AI were indeed available. One aspect that evokes a wide range of opinions was the availability of input data. On both sides of the spectrum, the arguments (i.e., there is a satisfying vs. insufficient amount of data) did not appear to be fact-based and instead seemed to reflect personal attitudes. For example, the statement that medical data are unlikely to be machine-readable, because medical professionals are believed to have bad handwriting. However, a more substantial number of comments in this category focused on AI abilities that are superior to those of humans. These comments often focused on the reliability and accuracy of AI, but AI ability to process data was also frequently mentioned:

*[U9]: And in the long run, whereas as a radiology resident in training I may read for example 10[,]000 chest radiographs during my residency of 5 years, AI will ultimately do this within a minute. There will be no way to compete.*

Some medical professionals argued that these abilities would enable AI to outperform medical professionals, or at least less-qualified medical professionals. However, we also found more moderate voices that argued that AI only has to be as good as an average medical professional:

*[U10]: The technical hurdles of development are also surmountable. There need not be 100% accuracy[; rather, AI only needs to be] just as good as the average radiologist[,] who is, herself, imperfect. The autonomous vehicle analogy is fitting here: we just have to be as good as the average driver, but there is the potential to be better.*

Apart from these more general discussions of the performance of medical AI in comparison with the performance of medical professionals, we also found lengthy and knowledgeable discussions about specific medical procedures that AI could augment and improve. Here, the ideas focused mainly on imaging-related procedures, such as grayscale imaging, contrast timing for imaging, and mammography. Other ideas included more comprehensive processes, such as triaging or even diagnostics. Interestingly, some medical professionals expressed the hope that AI could reduce the number of patients in urgent care:

*[U11]: It's going to quickly evolve to people asking "Alexa, what should I take for my headache?" and getting rational answers. Google search already does this. Try it. These technologies are going to [stop a lot of people from immediately going to see doctors in urgent-care environments].*

Another important aspect in the discussions involved opinions and attitudes towards AI acceptance, which again displayed a rich contrast of viewpoints. Several individuals viewed AI positively and were eager for AI to take over tedious tasks:

*[U12]: Personally, I would welcome not having to process 100 CXR a day and I don't know any radiologist that likes doing this. Radiology is 95% about slice imaging by now and I would actually prefer to spend my time getting good at stuff like ultrasound which actually nobody is good at where I work although it would be super useful.*

Several voices were skeptical about AI. A recurring argument compared AI’s use in medical fields with poorly functioning self-driving cars. Thus, automating medical procedures that are perceived as being more complicated than driving was viewed highly critically. This skepticism often also stemmed from the perception of current technological support, such as machine reads from EKGs, which remain inadequate. However, this argument was countered by other users:

*[U10]: Your analogy to EKG interpretation isn’t really fair. There is no financial or medical imperative to actually improve the E[K]G interpretation technology that exists today. […] If someone invested in […] the best EKG interpretation tools, and made it 100% perfect[,] it would not eliminate the need for emergency physicians or cardiologists.*

The criticism of AI appeared to center around the role of medical professionals. Indeed, medical professionals who rely on AI were described as lazy, or AI was described as a crutch for medical professionals with weak clinical performance. Others felt that the focus on AI’s takeover of medical professionals' roles was due to a “public hate” for these medical professionals. Interestingly, references to the role of medical professionals often appeared in calls for a more reasonable discussion and involved the assumption that medical professionals are biased due to their emotional investment in the field:

[U13]: It’s natural for us to try to hold on [to] our pride that certain things can never be learned by cold hard machines, […] but Watson and AlphaGo are perfect examples that it’s not about *if* we will be beaten in anything, but *when* .

Some of these comments, however, appear less objective and indicate that medical professionals are scared of AI or are worried that they have spent so many years studying only to be replaced. These commenters (which also came from non-medical professionals, e.g., AI developers) assumed that most comments that were skeptical about AI success had only been made by medical professionals to comfort themselves. While some medical professionals tended not to voice reservations about working with AI, a great deal of attention was given to these medical professionals’ lack of trust in AI’s ability to take care of patients. Medical professionals perceived this AI problem as an argument that were similar to their assumptions about patients’ reactions to AI above. The main fear was that any mistakes or technical issues with AI could have severe consequences for patients. To put it more drastically:

*[U14]: A "highly confident entirely wrong answer" can kill somebody.*

#### 3.2.5 A proposed way forward

Alongside discussions about both the potential of AI to replace medical professionals and the expected relationship between these medical professionals and AI, we found threads that aimed to establish requirements for using AI. One of the prominent sentiments was a call for a factual discussion about AI moving forward:

[U15]: The main issue is [that] the news continually sensationalizes *artificial intelligence* to be more than it actually is. It's just applied statistics and math.

Another important point was the call for better evaluations of AI applications, for example, through the involvement of medical professionals, through additional quality assurance steps, or through extended tests under real-life conditions. Recommendations spanned the whole AI lifecycle and ranged from the initial planning of technology in terms of providing a wider selection of AI use cases with a focus on value (e.g., in terms of significant workload reduction, performance, and health outcomes) on the one hand to assuring proper integration and adequate maintenance until the end of the AI lifecycle on the other hand. Even medical professionals with a positive perspective on the potential of AI agreed that AI still requires technical advancements, such as advancements in natural language processing in order to cope with patient input or existing clinical documentation. These challenges require medical professionals and AI specialists to collaborate, especially due to the substantial changes to the provision of future care:

*[U10]: I do believe AI will put radiology up first on the [chopping] block. Therefore, working together to understand this issue could prevent us from making the mistakes of the past.*

Such a collaboration requires both sides to deepen their knowledge of AI as well as of medicine and medical processes. For example, medical professionals need to learn about AI, and technological developers need to take into account physicians’ requirements in terms of their daily routines and ideals. Several threads included calls to incorporate AI-relevant skills into medical curricula and to engage medical students and professionals alike with AI-related content, which would enable these individuals to also integrate AI into practice. We read many low-threshold offers from the community for practitioners seeking a starting point:

*[U16]: I'd subscribe to the daily newsletter, AI in Healthcare[,] where most of the articles relate to imaging. Also, the websites of those companies that have FDA-cleared AI algorithms like Aidoc, Zebra, VizAI, MaxQ,* etc. *have good research articles based on AI impact on productivity and quality.*

Finally, a number of threads discussed the thoughts of medical professionals about AI research. An overarching theme involved discussions of AI research methods and the reported results. Medical professionals felt that AI studies that had been conducted in extremely controlled environments could not be translated into practice. In that regard, these medical professionals came to the conclusion that the field is still in its infancy:

*[U17]: Currently we get a lot of articles suggesting [that] medical AI systems are going to replace doctors or disrupt medical practice. I argue that almost all of this research is actually stuck [in] the equivalent of phase I trials, and thus quite a long way off [from] being ready for patients and clinics.*

Medical professionals frequently emphasized that AI research tends to exaggerate its findings and does not make sufficiently substantial contributions and implications for applications in the field, for example, because it does not address relevant tasks:

*[U18]: That said, the computer does not outperform human doctors [in] predicting heart attack[s]. CVD risk [does] not=heart attack risk. Although MI is a subset of CVD, it is not that specific. 22 factors is still far too little to predict [a] heart attack accurately. Plus[,] the difference between [the] ACA/AHA guidelines and the algorithm I think was 72–74% to 72–76%. A difference? Yes. A statistically significant one on paper? Yes. A clinically significant one? Maybe[,] who knows. What is more interesting to me is that despite almost a 3-fold increase in variable parameters, the computer was still not that much better than […] the guideline.*

In line with this sentiment, medical professionals also discussed the need for a stronger collaboration with AI developers in order to tackle relevant problems that are not (yet) able to be solved with current AI methods.

### 3.3 Discussion

To explore opinions and attitudes towards the use of AI among health care professionals, we qualitatively examined the subreddits social media platforms that exclusively focus on medical profession, i.e., r/medicine, r/radiology, r/surgery, and r/psychiatry. Our findings suggest that medical professionals are mainly concerned with three major and distinct themes in the context of AI adoption: consequences, the current state of AI in the medical field and a proposed way forward.

Among the themes, the most prominent topic that we found involved discussions about job-replacement anxiety, i.e., the fear that a medical professionals’ job will be replaced by AI. Discussants represent opinions that AI may completely replace some healthcare jobs in the future, AI is a good tool to augment (or support) medical professionals, and the denial that AI could ever replace human physicians. Regarding the potential replacement of medical professionals, some participants of the discussion argued that physicians are biased against AI and its adoption because they feel threatened. What we found was that the medical professionals often focused on the current way of doing things in their arguments. Thus, discussions centered around aspects of the current practices that AI cannot support or improve, e.g., applying common sense to detect if patients were accurate in reporting their medical history.

The tendency of taking the present state as a reference point and evaluating the outcomes of other alternatives as disadvantageous (losses) or advantageous (gains) changes from this reference point is a common behavioral pattern observed in human decision making. Subjectively, the losses of leaving the current state loom larger than the gains [45] [45] and people, therefore, tend to remain in the current state even if it would be advantageous to leave it. The tendency to prefer the status quo over an advantageous alternative is commonly known as the status quo bias [45], [73]. The status quo bias has repeatedly been linked to negative attitudes towards new technologies, slow technology adaption, and resistance against using innovative information systems [12], [37], [36], [47], [64]. Thus, in addition to job replacement anxiety, the status quo bias may influence attitudes and opinions toward AI application in health care and it may diminish AI-adoption and intention to use AI among medical professionals.

In line with prior literature, we found that medical professionals tend to justify a critical perspective on AI for medical use by referring to the insufficiencies of AI in different domains, e.g., failures of self-driving cars. However, when arguments became perceptibly unobjective, other participants typically called these arguments out as being not fact-based, and the discussion tended to return to a more meaningful exchange of ideas. The variety of discussed options highlights the relevance of the exchanges that take place on platforms such as Reddit when it comes to ascertaining the full effects that AI could have on the practice of medicine. These insights also have practical relevance. Based, for example, on the differences in the perceived value of full automation or augmentation by AI, technical solutions should allow the user to decide the level of desired support by the medical AI. But the discussions on reddit also highlight the importance of medical professionals to engage in these debates to keep discussions on a fact-based level and to avoid negative influences on technology acceptance by exaggerated representations. However, these discussions also need to happen offline to ensure that the needs of everybody involved are addressed, e.g., through dedicated trainings which can resolve user resistance [42].

Another important aspect of the discussions that we analyzed was the medical professionals’ critical reflection on AI research. The physicians whose statements we analyzed questioned the extent to which study designs had only chosen to showcase the strengths of AI as well as the extent to which the results of these studies would hold up in a real-life setting. Such reflection is necessary in order to keep expectations realistic and to avoid exaggerating results because evaluation processes for medical AI are still limited [87]. However, some of the posts implied that AI research is generally not to be trusted, for example, due to financial interests in exaggerating results. Such general mistrust could be worrisome as it could hamper a fact-based discussion of potential uses of medical AI. No specific research has yet been conducted on the quality of medical AI research. While general concerns about biases in medical research – including reporting bias [55], gender bias [28], and volunteer bias [19] – are known, these biases appear to be insufficient to generally discredit AI research. Our findings underline the need to further improve research standards and to communicate result limitations even more clearly.

In summary, the findings show that opinions and attitudes towards using AI technology in healthcare are mixed among medical professionals. Some of them showed a progressive attitude in this context, i.e., a positive view on using AI in their jobs. However, job-replacement anxiety seems to be a prominent concern among medical professionals. The fear of being replaced was a frequently discussed topic in the subreddits, often characterized by a negative attitude towards implementing AI technology in clinical tasks. To shed light on the ambiguous opinions and attitudes towards using AI that we have found among medical professionals in our first study, we conduct a second study to investigate the willingness of medical professionals to adopt AI technologies in clinical tasks and the role of job-replacement anxiety as well as AI knowledge in this context in more detail.

## 4 Study 2

In study 2, we used a cross-sectional research approach by conducting an online survey to investigate the impact of the fear of medical professionals that their jobs will be replaced by an AI on the intention to use AI technology. We further included a measure of domain-specific knowledge about AI since previous research as well as our results from the first study suggest that little or mixed knowledge levels of medical professionals seem to shape their picture of AI and its consequences. Particularly, research has shown that a lack of knowledge about AI technologies can enhance the fear of it, and that individuals with greater AI knowledge have more positive attitudes towards AI-enabled technologies [40] which may, therefore, attenuate the intention to use it. Building upon the findings of our first study, empirical evidence from previous work and theoretical considerations outlined in the previous chapters, we derive the following hypotheses:

H1 *The intention to use AI technology is decreasing among medical professionals with increasing anxiety that AI will replace their job.*

H2 *Medical professionals’ intention to use AI technology increases with increasing domain-specific knowledge about AI.*

H3 *Increasing domain-specific knowledge mitigates the effect of job-replacement anxiety on the intention to use AI among medical professionals.*

Furthermore, we exploratively investigate how other dimensions of AI-related anxiety, based on the proposed scale by Wang and Wang [85] affect the intention to use AI. Their scale proposes that people are also scared of learning about AI (similar to statistics), the humanoid appearance (i.e., AI configuration), and the possible consequences of misuse of AI and autonomous AI (i.e., sociotechnical blindness). As these dimensions were also inherently reflected in our discovered themes of study 1, we included these subscales in this study. The study was conducted using Amazon MTurk, Prolific and the online survey software EFS from TIVIAN.

### 4.1 Method

#### 4.1.1 Materials, design, and procedure

To test our hypotheses, we collected self-reported data from medical professionals using an online survey. This study was part of a larger study investigating the impact of AI advice on human behavior. In this section, we only report the information and results relevant to this article. First, participants received basic information about the study and were invited to participate voluntarily. Furthermore, they were informed that we added attention checks to the measures where participants were asked to give a particular rating (e.g., “please rate this item with four") and that an attention check failure will terminate the study. After participants have agreed to the informed consent form, they were introduced in the survey procedure. They then responded to several survey items and measures. Finally, they were asked to provide some demographic information and the frequency of using AI in their job. On the last survey page, participants received an individual, randomly generated code required to get the participation fee.

#### 4.1.2 Measures

We included an adapted a version of the intention to use measure [2], [4], [82] as the dependent variable. The scale includes two items measuring the intention to use AI and the intention to increase the use of AI on a 7-point-Likert-scale ranging from 1 (strongly disagree) to 7 (strongly agree). The specific items of the scale can be found in Appendix B. We observed a Spearman-Brown reliability estimate of the intention to scale of 0.65. Note that the Spearman-Brown coefficient is the preferable reliability estimate for scales including two items [29].

We further included two explanatory variables: The artificial intelligence anxiety. Scale [85] and an adapted version of the domain-specific consumer knowledge scale [51], [57], [84].

The artificial intelligence anxiety scale includes 21 items in four subscales (“dimensions”). The subscales address AI anxiety in the context of learning (8 items, e.g., “Learning to use AI techniques/products makes me anxious”), job-replacement (6 items, e.g., “I am afraid that AI techniques/products will replace someone’s job”), sociotechnical blindness (4 items, e.g., “I am afraid that an AI technique/product may be misused”), and AI configuration (3 items, e.g., “I find humanoid AI techniques/products (e.g., humanoid robots) scary”). Participants rated each item using a 7-point Likert-scale ranging from strongly disagree to strongly agree.^1^ We observed reliabilities of α = .92, α = .88, α = .82, and α = .77 for the learning, job-replacement, sociotechnical blindness, and AI configuration subscales, respectively. All 21 items of the artificial intelligence anxiety scale are shown in Appendix B.

The domain-specific consumer knowledge scale included 7 items. For each item, participants self-report their knowledge about AI technologies compared to “the average person” using a 7-point-Likert-scale ranging from 1 (strongly disagree) to 7 (strongly agree). For example, one item is “I am very familiar with AI-based technology”. We observed a reliability of α = .69. The complete domain-specific consumer knowledge scale can be found in Appendix B.

#### 4.1.3 Participants

We requested participants on Amazon MTurk and Prolific. They were prescreened according to their employment sector. In particular, participants were required to work as physician, nurse, paramedic, and/or in the emergency medical services. They received a hyperlink that directed to the online survey. The online experiment was open for participation on Amazon MTurk and Prolific for the last two weeks in October 2023. Participants were not required to meet any additional qualifications to participate (i.e., minimum HIT approval rate, language, location). 540 individuals accepted the participation request (524 on MTurk, and 16 on Prolific). In total, 223 (118 female, 103 male, 2 preferred not to say) participants completed the survey. The mean age was 32.7 years (median: 35, range: 23–70, SD: 6.8, n = 6 preferred not to say). Participants gave their informed consent prior to their inclusion in the study. Completion time was 14 min and 39 s on average. Participants received a compensation of $2.11 / £ 1.74.

#### 4.1.4 Data analysis

We normalized the values of the scales by subtracting the smallest measurable value of the measure (*I*_*mi*n_) from the value recorded for each participant (*I*_*i*_) and divide the result by the highest measurable value of the instrument (*I*_*max*_) minus *I*_*min*_:

*I*_*norm*_*=* $\frac{I_{i}-I_{\min\;}}{I_{\max}-I_{\min}}$*.* This was done to improve comparability, standardization, and interpretability of scores with different ranges. Normalizing values of different measures is a common practice in psychological research (see, e.g., [26], [89]). We analyzed the data using descriptive statistics, Pearson correlations, and generalized linear models (main effects and interactions). We used the computing environment R for statistical evaluation (version 4.2.0; packages: “descr”, “psych”, and “Hmisc”; [7], [38], [66], [70]).

### 4.2 Results

16 participants dropped out, because they indicated an occupation not meeting the participation requirements. We further excluded data from 301 participants who failed an attention check. We included data provided by the remaining 223 participants who completed the survey. Table 3 shows the correlations between the measures we used in the current study. We observe several significant positive correlations between the scales. In particular, intention to use AI correlates with domain-specific consumer knowledge, the AI Anxiety sub-scales and the frequency the participants use AI techniques in their jobs. Furthermore, domain-specific consumer knowledge correlates with AI Anxiety Learning subscale and frequency of using AI in Job, and AI Anxiety Learning correlates with frequency of using AI in Job. Moreover, all the AI anxiety subscales correlate with each other, which is, of course, not surprising. However, we observe small (r > .1), moderate (r > .3) and large effect (r > .5) effect sizes [22].

**Table 3:** Matrix of Pearson correlations between the measures.

	ITU	DSCK	AIAJ	AIAL	AIAC	AIAS
DSCK	**0.57**					
*p*	*< 0.001*					
AIAJ	**0.34**	0.01				
*p*	*< 0.001*	*0.863*				
AIAL	**0.39**	**0.13**	**0.78**			
*p*	*< 0.001*	*0.048*	*< 0.001*			
AIAC	**0.36**	0.05	**0.71**	**0.77**		
*p*	*< 0.001*	*0.496*	*< 0.001*	*< 0.001*		
AIAS	**0.33**	0.00	**0.84**	**0.74**	**0.70**	
*p*	*< 0.001*	*0.971*	*< 0.001*	*< 0.001*	*< 0.001*	
AI use	**0.19**	**0.31**	0.06	**0.17**	0.00	0.07
*p*	*0.005*	*0.000*	*0.361*	*0.012*	*0.988*	*0.315*

Note. ITU: Intention to use AI, DSCK: Domain-specific consumer knowledge, AIAL: AI Anxiety Learning, AIAJ: AI Anxiety Job-replacement, AIAC: AI Anxiety Configuration, AIAS: AI Anxiety Sociotechnical blindness, AI use: Frequency of using AI in job. Statistically significant correlations (p < 0.05) are printed in bold, and p-values are italicized.

To test the hypotheses that propose a direct relationship between the dependent variable “intention to use AI” and the explanatory variables “AI Anxiety Job-replacement” (H1) and “Domain-specific consumer knowledge” (H2), we conducted a linear regression analysis (Table 4, main effects model). In addition, we also include the other subscales of the AI anxiety scale for exploratory evaluation. The analysis showed that domain-specific consumer knowledge about AI significantly predicts intention to use AI: The intention to use AI increases among medical professionals with increasing knowledge about AI. However, the linear regression analysis of the main effects did not indicate a significant impact of the AI anxiety subscales on the intention to use AI.

**Table 4:** Linear regression models: main effects model and interaction effects model.

	Main-effects model					Interaction-effects model			
	Est.	SE	t-value	p-value	Est.		SE	t-value	p-value
AI Knowledge	0.674	0.062	10.857	< 0.001 * **	0.872		0.173	5.028	< 0.001***
AI Anxiety (job replacement)	0.091	0.098	0.928	0.354	-0.758		0.403	-1.880	0.062
AI Anxiety (learning)	0.030	0.084	0.358	0.720	0.311		0.314	0.989	0.324
AI Anxiety (configuration)	0.111	0.093	1.194	0.234	0.133		0.370	0.360	0.719
AI Anxiety (soc.tech. blindness)	0.147	0.075	1.957	0.052	0.943		0.426	2.214	0.028*
AI Knowledge x AI Anxiety (job)					1.424		0.670	2.124	0.035*
AI Knowl. x AI Anxiety (learning)					-0.421		0.497	-0.847	0.398
AI Knowl. x AI Anxiety (config.)					-0.042		0.613	-0.068	0.945
AI Knowl. x AI Anxiety (soc.tech)					-1.344		0.681	-1.973	0.049*
(Intercept)	0.031	0.048	0.649	0.517	-0.099		0.111	-0.887	0.376

Note. Main-effects model fit: F(5215) = 36.77, p < 0.001, R² = 0.46, interaction-effects model fit: F(9211) = 21.30, p < 0.001, R² = 0.48.

* p > 0.05,

*** p < 0.001.

The fact that our results did not show an effect of the AI anxiety subscales makes our third hypothesis redundant. However, the correlations between AI anxiety subscales and intention to use AI indicate a moderating role of particular AI anxiety subscales in the relationship between AI knowledge and intention to use AI. Therefore, we exploratory investigated interactions between the effects of AI knowledge and AI anxiety on the intention to use AI among medical professionals by performing a second linear regression analysis where the main effects linear model additionally incorporates the interactions between AI knowledge and the AI anxiety subscales.

**Fig. 2:**
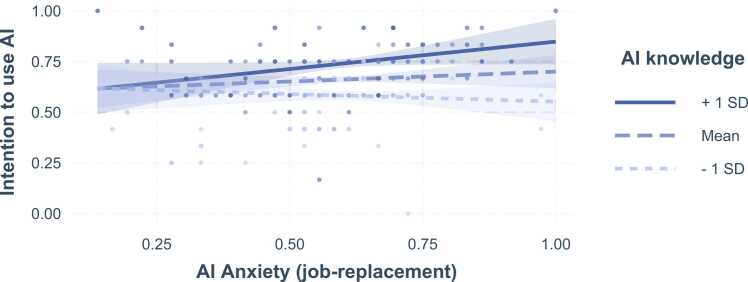
Interaction effects. AI knowledge by AI Anxiety (job-replacement). Note. Areas around the graphs indicate 95% confidence intervals. Points represent actual data, color of the points indicates AI knowledge.

We found that the AI anxiety subscales of job-replacement and sociotechnical blindness (i.e., the fear that AI may get out of control or may be misused) significantly moderate the impact of AI knowledge on medical professionals’ intention to use AI. In particular, stronger AI anxiety (job-replacement) enhances the effect of AI knowledge on intention to use AI. That is, the impact of AI knowledge on the intention to use AI increases for medical professionals that have a greater fear that AI may replace jobs. As illustrated in Fig. 2, for medical professionals with AI knowledge one standard deviation below the average of the sample, the intention to use AI slightly decreases with increasing AI anxiety (job-replacement). For those with greater AI knowledge (one SD above the mean), this effect is reversed, i.e., the intention to use AI increases with increasing job replacement AI anxiety.

**Fig. 3:**
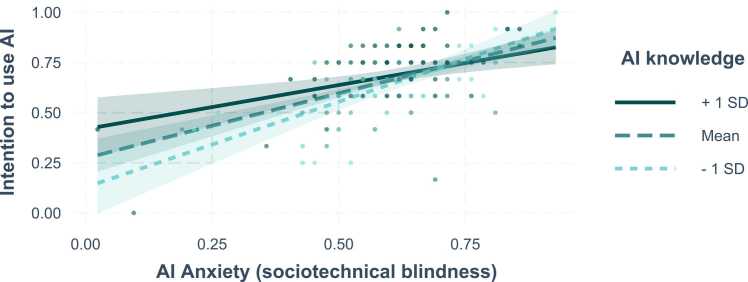
Interaction effects. AI knowledge by AI Anxiety (sociotechnical blindness). Note. Areas around the graphs indicate 95% confidence intervals. Points represent actual data, color of the points indicates AI knowledge.

We observe the opposite effect for the interaction of AI knowledge by AI anxiety (sociotechnical blindness),: For medical professional with lower scores of AI anxiety (sociotechnical blindness), the intention to use AI increases with AI knowledge. This effect, however, seems to disappear with increasing scores of this type of AI anxiety (see Fig. 3). Interestingly, we further observed the tendency that the intention to use AI increases with increasing AI anxiety (sociotechnical blindness). This tendency seems to be most prevalent for participants with lower AI knowledge (one SD below the average) compared to the average (mean SD) and to those with higher AI knowledge (one SD above the mean).

Finally, we adjusted the regression models for some demographic information of the participants, i.e., age, sex, and education by analyzing a potential impact of these variables on the dependent variable, i.e., intention to use AI, using linear regression analysis. No significant effects were found. The model fit parameters were F(3,217) = 1.07, p = 0.36, and R² = 0.01).

### 4.3 Discussion

In study 2, we systematically investigated the interrelations between AI anxiety, domain-specific knowledge of AI, and the intention to use AI among medical professionals. This inquiry is particularly pertinent in the context of the evolving landscape of AI in healthcare, where its implications for medical professionals are subjects of significant debate. In examining hypotheses, our study did not find an inverse relationship between job-replacement anxiety and the willingness of medical professionals to use AI technology (H1). This is particularly revealing, as it is seemingly a prevalent concern in the healthcare sector. Furthermore, the anxiety is not unfounded, given the rapid advancements in AI capabilities, which often lead to speculation about the automation of tasks currently performed by human professionals. However, it is essential to contextualize these findings within the broader landscape of healthcare, where the integration of AI is not merely a matter of replacing human labor but rather augmenting and enhancing the capabilities of medical professionals. Based on the specific AI-enabled technology and also the field of application (e.g., psychiatry or radiology) there might be differences [41]. Hence, a nuanced understanding is still crucial for developing strategies to mitigate such anxieties, which, if left unaddressed, could significantly hinder the adoption and optimal utilization of AI in healthcare settings.

The positive correlation identified in H2 between domain-specific AI knowledge and the intention to use AI in medical practice offers an optimistic view with regard to AI adoption as well as to the anxieties surrounding AI. This finding aligns with previous research [40], [8] and our discussions of study 1. In the context of AI in healthcare, this suggests that when medical professionals are equipped with a deeper understanding of AI (e.g., including its practical applications, limitations, and ethical considerations) they are more likely to perceive it as a beneficial tool and intend to use it. This insight is pivotal for healthcare administrators and educators, highlighting the need for comprehensive AI education programs that go beyond mere technical training to include ethical, practical, and collaborative aspects of AI in healthcare.

H3 explored the potential moderating effect of AI knowledge on the relationship between job-replacement anxiety and the intention to use AI. However, as H1 was not supported, the implications of these results should be interpreted with caution. The observation in our analysis provides a further argument for the role of education in mitigating AI-related anxieties. Particularly, the finding suggests that when medical professionals possess a substantial understanding of AI, their concerns about job security diminish, and their openness to using AI increases. This could be attributed to a more informed perspective on how AI can act as a complement to, rather than a replacement for, their professional expertise. For instance, AI's role in diagnostic processes can be viewed as augmentative, enhancing the accuracy and efficiency of diagnoses while still relying on the critical judgment and contextual understanding of medical professionals. This perspective shift, facilitated by increased knowledge, is crucial for fostering a more positive attitude towards AI adoption in healthcare.

The effect of knowledge was also present in our further explorative analysis. With regard to anxiety in terms of sociotechnical blindness (e.g., misuse of AI or autonomous), the effect could only be observed for medical professionals with lower levels of anxiety. The findings suggest, that when this type of anxiety exceeds a certain threshold even knowledge cannot mitigate the unwillingness to use AI. This seems plausible as perceptions of a real strong AI [60] could really lead to full automation. However, with regard to other forms of AI anxiety (i.e., learning and AI configuration) no effect of knowledge was observed, suggesting that research should have a nuanced view on anxiety about AI.

## 5 General discussion

Generally, AI holds significant potential for medical practice [90]. However, similar to other domains, the acceptance of AI is crucial in order to enable the full value of the technology to be reached. A negative perception among involved stakeholders – especially among the medical professionals who must interact closely with AI – may inhibit the intention to use AI-enabled technology despite its potential benefits.

Against this background, our research sheds light on the research question what medical professionals think about adopting AI-enabled healthcare technologies and contribute to the literature of AI adoption [49], [88]. To answer our RQ, we conducted two consecutive studies. In our first study, we gathered an understanding what medical professionals think about AI in general and identified three reflecting key themes: (1) perceptions of the status quo of the technological environment and of the consequences of AI, (2) the physician–AI relationship, and (3) a proposed way forward. Generally, we observed mixed statements about AI. In addition to positive attitudes towards using AI-enabled technology in healthcare, we also found negative ones in the statements, often associated with the fear of being replaced by an AI and little knowledge about AI. Moreover, the intention to use innovative technology such as AI might be inhibited by systematic mental errors in judgment and decision-making (i.e., cognitive biases; [81]). One bias that have been shown to affect both medical decision-making [17] and acceptance of innovative technology [33] is the so-called status-quo bias. The status-quo bias highlights the natural human tendency to cling to familiar practices and systems, even in the face of potentially beneficial innovations [45], [73]. This bias is a significant barrier to AI adoption, as it often leads to an overemphasis on the limitations of AI [12], [37], [36], [47], [64], while underappreciating its potential benefits. Thus, an unbiased and open-minded intention to use requires more than just technological advancements; it calls for a change in mindset and a cultural shift within the medical (AI) community. As our results suggest, not all medical professionals possess this view yet.

Based on the findings of the first study, we dived deeper to gather a more nuanced understanding how anxiety about AI and knowledge about AI influences the intention to use among medical professionals. The results highlight that anxiety were not as evident as suggested by our qualitative analysis in study 1. Contrary to our findings of the first study and prior research [1], [33], [41], [61], we found no inverse relationship between AI-related job replacement anxiety and the willingness to use AI technology. That is, although anxiety about AI is used as a tool for augmentation against its implementation in healthcare, it does not seem to immediately affect the intention to use AI. This might be attributed to our study's approach, which analyzed the impact of AI-based systems at a holistic level. Interestingly, similar results have been observed in recent research concerning general attitudes towards AI, indicating a broader trend [46]. However, Huo et al. [41] investigated the impact of AI-anxiety on medical AI for independent and assistive diagnosis and treatment, separately. They found the acceptance of AI for assistive diagnosis and treatment technology decreasing with increasing AI-anxiety, and no significant relationship between AI-anxiety and independent diagnosis and treatment AI-systems. Nevertheless, our second study underscores the importance of domain-specific AI knowledge in shaping attitudes towards technology adoption. The positive correlation between AI knowledge and the willingness to use AI suggests that education and knowledge dissemination could be crucial in engaging medical professionals to make use of innovations.

Generally, our findings reiterate the relevance of the opinions and attitudes towards AI among medical because they could not only foster adoption among medical professionals but furthermore have a significant influence on patient’s perceptions of the technology. The patient-physician relationship is especially important as previous studies in the medical field highlight the influence third parties can have on the technology acceptance of patients [48]. To ensure that medical professionals not only adopt but also can wholeheartedly recommend AI solutions, a comprehensive approach addressing multiple aspects is needed. For instance, the educational aspects of AI must be comprehensively addressed, encompassing ethical, practical, and collaborative dimensions. Furthermore, in the context of AI development, ensuring the quality of care must be a fundamental and clearly articulated principle guiding the design of medical AI applications. Furthermore, these findings should encourage all stakeholders among physicians, patients, healthcare management, and AI researchers and developers to do their best to encourage a fact-based, constructive discussion shaping realistic expectations and judgements about AI. In this line, AI research should also be more carefully framed in order to avoid overpromising results and impact. Finally, open discussions between medical professionals and patients about possible AI use should be promoted in order to prevent the mistrust of potentially helpful technology among medical professionals.

## 6 Limitations

We would like to remark that our studies have the following limitations. First, as subreddits are anonymous, we could not reliably determine the profession of the post authors in study 1. The same applies to the information provided by the survey participants in study 2. Being able to distinguish between, e.g., medical students, physicians in a clinical setting, physicians with their own practice, and AI researchers would have allowed us to examine potential differences between different groups with different viewpoints and experiences. However, in comparison with prior work, we provided a more specific picture of the perceptions of AI in medicine due to our analysis of medicine-focused subreddits as well as a consecutive online survey of healthcare workers. Second, the use of subreddits is subject to a self-selection bias, as users who are more open and tech-savvy [35], for example, are more likely to participate here. Research also indicates that data from Reddit is subject to further biases such as gender or language bias [32], [54]. Similar applies for the online survey as participants are online users who self-select tasks [3]. Therefore, generalizability is limited. However, as the results come from rather technology-savvy, open users, the results should raise awareness and encourage further investigations. Particularly, future research could benefit from further empirical insights from a field study with a diverse sample of medical professionals.

Finally, we used a comparatively small dataset for LDA, and the data used was based on a heuristic filtering method (i.e. Wikipedia's AI glossary), which may not have included all the data relevant to the analysis. However, by combining quantitative and qualitative methods, we were able to significantly mitigate the risk of arriving at incorrect conclusions. Nevertheless, we could only observe the thoughts and opinions shared within the posts. As mentioned before, future research may provide additional field study data – for example, obtained from interviews – that could yield deeper insights into the reasoning behind these thoughts.

## 7 Conclusion

Healthcare systems around the world are currently facing immense challenges [25], [79] that digital technologies – and especially medical AI – could help to solve [58], [83]. However, the value that AI prototypes have demonstrated in various clinical tasks [90] might not yet have been fully captured due to both physicians’ resistance to using the technology and to the lack of acceptance of AI among these physicians [43]. Our social media analysis of physicians’ perceptions of AI in medical subreddits revealed three key themes: (1) perceptions of the status quo of the technological environment and of the consequences of AI, (2) the physician–AI relationship, and (3) a proposed way forward. The first and the second theme, in particular, appear to have been affected by biased behavior. Considering that the sample of perceptions of AI are originating from medical professionals using Reddit, which indicates a higher technological affinity compared to the overall population, the results have important implications. These findings should inform and help all stakeholders involved in the introduction of medical AI technology to address perceptions and accompanying issues more consciously in order to help prevent resistance to AI in the medical field. To address these issues, our second study furthermore highlights the critical role of domain-specific AI knowledge in shaping medical professionals' intention to use AI technology. By mitigating job-replacement anxiety, such knowledge not only fosters a more positive attitude towards AI but also paves the way for its more effective integration into healthcare practices. As the landscape of AI continues to evolve, ongoing education and dialogue will be key in harnessing its potential in a way that complements and enhances the invaluable work of medical professionals.

## CRediT authorship contribution statement

**Sebastian Weber:** Conceptualization, Methodology, Validation, Software, Data Curation, Investigation, Formal analysis, Writing- Original draft, Writing - Review & Editing, Visualization, Project administration. **Marc Wyszynski:** Data curation, Writing- Original draft, Writing - Review & Editing, Methodology, Investigation, Formal analysis, Visualization. **Marie Godefroid**: Conceptualization, Formal analysis, Validation, Data Curation, Writing - Original Draft. **Ralf Plattfaut:** Supervision, Conceptualization. **Bjoern Niehaves**: Supervision, Conceptualization, Resources.

## Declaration of Competing Interest

None declared.

## Figures and Tables

**Table:** 

Theme	Marginal topic distribution		Top 30 most salient terms within topics	
	3-topic	7-topic	3-topic	7-topic
1. Consequences of AI	1 (61%)	1 (64%)	ai, radiologist, would, think, well, make, get, patient, go, radiology, work, take, need, job, human, like, see, one, even, use, replace, say, people, medicine, year, time, could, read, doctor, computer	ai, radiologist, would, think, radiology, well, get, make, work, job, go, like, time, patient, take, say, could, see, use, good, one, read, need, human, even, replace, people, year, image, also
2. Physician–AI relationship	2 (25%)	2 (15%)	ai, radiologist, would, work, think, well, get, like, go, use, say, time, one, make, radiology, doctor, patient, medicine, job, good, year, could, see, computer, know, physician, people, read, take, much	patient, ai, work, get, doctor, go, think, make, would, take, well, year, like, medicine, need, lot, people, one, physician, want, know, also, good, job, see, thing, time, use, say, could
3. A proposed way forward	3 (14%)	3 (11%)	patient, use, think, good, would, work, one, could, see, thank, well, make, time, get, like, know, also, want, find, say, need, really, suture, ai, model, system, scan, lot, go, look	ai, would, use, work, well, need, computer, diagnosis, make, human, patient, go, datum, think, see, disease, lot, ml, thing, even, like, medicine, one, model, base, know, clinical, algorithm, machine, could
	-	4 (4%)	-	pneumonia, algorithm, paper, radiologist, computer, use, study, data, set, ECG, say, cardiologist, well, ml, arrhythmia, performance, system, diagnosis, diagnose, one, gold_standard, like, label, look, chexnet, claim, test, author, read, compare, identify
		5 (4%)		patient, use, thank, one, model, risk, system, find, method, think, start, study, disease, algorithm, COVID, care, lung, research, datum, population, good, target, also, new, make, specific, paper, predict, without, symptom
		6 (2%)		datum, fracture, use, image, bone, deep_learne, detection, software, ml, pe_ct, dataset, watson, one, mole, ct, look, head_ct, segmentation, slice, pneumothorax, pathologist, post, product, real_world, stroke, picture, problem, google, segment, positivity
		7 (<1%)		aspire, eye, spade, oct, experiment, retina, nonsensical, prime, creep, instant, diagram, prioritize, macular, triaging, helping, fulfill, elapse, degeneration, tire, sufferer, pile, revise, redo, outlines, declare, divorce, concurrent, tenacity, immigrant, pinnacle

## Appendix B Measurement instruments

### Intention to use AI

1. Assuming that I have access to AI-based technologies, I intend to use them.
2. I intend to increase my use of AI-based technologies in the future.

### Artificial intelligence anxiety scale

#### Learning subscale

1. Learning to understand all of the special functions associated with an AI technique/product makes me anxious.
2. Learning to use AI techniques/products makes me anxious.
3. Learning to use specific functions of an AI technique/product makes me anxious.
4. Learning how an AI technique/product works makes me anxious.
5. Learning to interact with an AI technique/product makes me anxious.
6. Taking a class about the development of AI techniques/products makes me anxious.
7. Reading an AI technique/product manual makes me anxious.
8. Being unable to keep up with the advances associated with AI techniques/products makes me anxious.

#### Job-replacement subscale

1. I am afraid that an AI technique/product may make us dependent.
2. I am afraid that an AI technique/product may make us even lazier.
3. I am afraid that an AI technique/product may replace humans.
4. I am afraid that widespread use of humanoid robots will take jobs away from people.
5. I am afraid that if I begin to use AI techniques/products I will become dependent upon them and lose some of my reasoning skills.
6. I am afraid that AI techniques/products will replace someone’s job.

#### Sociotechnical blindness subscale

1. I am afraid that an AI technique/product may be misused.
2. I am afraid of various problems potentially associated with an AI technique/product.
3. I am afraid that an AI technique/product may get out of control and malfunction.
4. I am afraid that an AI technique/product may lead to robot autonomy.

#### AI configuration subscale

1. I find humanoid AI techniques/products (e.g., humanoid robots) scary.
2. I find humanoid AI techniques/products (e.g., humanoid robots) intimidating.
3. I don’t know why, but humanoid AI techniques/products (e.g., humanoid robots) scare me.

### Domain-specific consumer knowledge

Compared to the average person….

1. … I do not know much about AI-based technology. (-)
2. … I am very familiar with AI-based technology.
3. … I am not knowledgeable about AI-based technology. (-)
4. … I am very interested in AI-based technology.
5. … I use AI-based technology a lot.
6. … My colleagues use various AI-based technology.
7. … I read articles about AI-based technology all the time.

*Note:* All items were measured on a seven-point Likert scale (see study 2 – chapter 4.2 Measures for more information).

## Footnotes

1 Unfortunately, Y.-Y. Wang & Wang [85] did not provide information about the labeling of the Likert-scale values. Thus, we adopted the labels of the other scales included in this study to have a unified response architecture.
